# Longitudinal stability of asthma characteristics and biomarkers from the Airways Disease Endotyping for Personalized Therapeutics (ADEPT) study

**DOI:** 10.1186/s12931-016-0360-5

**Published:** 2016-04-23

**Authors:** P. E. Silkoff, M. Laviolette, D. Singh, J. M. FitzGerald, S. Kelsen, V. Backer, C. Porsbjerg, P. O. Girodet, P. Berger, J. N. Kline, S. Khatri, P. Chanez, V. S. Susulic, E. S. Barnathan, F. Baribaud, M. J. Loza, for the ADEPT Investigators

**Affiliations:** Janssen Research & Development LLC, Spring House, PA USA; Institut Universitaire de Cardiologie et Pneumologie de Québec (IUCPQ), 2725, Chemin Ste-Foy, Quebec, QC G1V 4G5 Canada; Centre for Respiratory Medicine and Allergy, the University of Manchester, Medicines Evaluation Unit, University Hospital of South Manchester NHS Foundation Trust, Southmoor Road, Manchester, M23 9QZ UK; Institute for Heart and Lung Health, The Lung Centre, Gordon and Leslie Diamond Health Care Centre, 7th Floor, 2775 Laurel Street, Vancouver, BC V5Z 1M9 Canada; Department of Thoracic Medicine and Surgery, Temple University School of Medicine, 401N. Broad St., Philadelphia, PA 19140 USA; Respiratory Research Unit, Department of Respiratory Medicine, Bispebjerg University Hospital, Bispebjerg bakke 23, DK-2400 Copenhagen, NV Denmark; Centre de Recherche Cardio-Thoracique de Bordeaux, University of Bordeaux, U1045, CIC 1401, F-33000 Bordeaux, France; Division of Pulmonary, Critical Care, and Occupational Medicine, University of Iowa, W219B GH UIHC, 200 Hawkins Drive, Iowa City, IA 52242 USA; Department of Pulmonary and Critical Care, Cleveland Clinic, 9500 Euclid Avenue, Cleveland, OH 44195 USA; Pneumologie, Aix Marseille University, APHM/INSERM U1067, Chemin des Bourellys 13015, Marseille, France; Present Address: 715 Bryn Mawr Avenue, Penn Valley, PA 19072 USA

**Keywords:** Asthma, Severity, Phenotypes, Profiling, Biomarkers, Longitudinal stability

## Abstract

**Background:**

Asthma is a biologically heterogeneous disease and development of novel therapeutics requires understanding of pathophysiologic phenotypes. There is uncertainty regarding the stability of clinical characteristics and biomarkers in asthma over time. This report presents the longitudinal stability over 12 months of clinical characteristics and clinically accessible biomarkers from ADEPT.

**Methods:**

Mild, moderate, and severe asthma subjects were assessed at 5 visits over 12 months. Assessments included patient questionnaires, spirometry, bronchodilator reversibility, fractional exhaled nitric oxide (FENO), and biomarkers measured in induced sputum.

**Results:**

Mild (*n* = 52), moderate (*n* = 55), and severe (*n* = 51) asthma cohorts were enrolled from North America and Western Europe. For all clinical characteristics and biomarkers, group mean data showed no significant change from visit to visit. However, individual data showed considerable variability. FEV1/FVC ratio showed excellent reproducibility while pre-bronchodilator FEV1 and FVC were only moderately reproducible. Of note bronchodilator FEV1 reversibility showed low reproducibility, with the nonreversible phenotype much more reproducible than the reversible phenotype. The 7-item asthma control questionnaire (ACQ7) demonstrated moderate reproducibility for the combined asthma cohorts, but the uncontrolled asthma phenotype (ACQ7 > 1.5) was inconstant in mild and moderate asthma but stable in severe asthma. FENO demonstrated good reproducibility, with the FENO-low phenotype (FENO < 35 ppb) more stable than the FENO-high phenotype (FENO ≥ 35 ppb). Induced sputum inflammatory phenotypes showed marked variability across the 3 sputum samples taken over 6 months.

**Conclusions:**

The ADEPT cohort showed group stability, individual stability in some parameters e.g. low FEV1/FVC ratio, and low FENO, but marked individual variability in other clinical characteristics and biomarkers e.g. type-2 biomarkers over 12 months. This variability is possibly related to seasonal variations in climate and allergen exposure, medication changes and acute exacerbations. The implications for patient selection strategies based on clinical biomarkers may be considerable.

## Background

Asthma is a heterogeneous disease where clinical and/or biomarker phenotyping increases the probability of success with novel therapies. Thus anti-IL13 monoclonal antibodies (mAb) are more efficacious in T helper 2 (Type-2) driven inflammation characterized by biomarkers e.g. periostin [1], fractional exhaled nitric oxide (FENO) [1], and dipeptidyl peptidase 4 [2]. The anti-interleukin 5 therapies are effective in those with elevated markers of eosinophilic inflammation in blood and sputum [3–6]. Similarly, an anti-IgE mAb, omalizumab, is more effective in patients with high periostin level, high FENO and high blood eosinophil count [7].

However, whether or not these phenotypic characteristics of asthma are stable in a single patient over time remains to be determined. Such a question is crucial since entry into a clinical trial, or selecting a therapy in the clinic, may be based on a single phenotypic assessment. Spontaneous variability, seasonal variation, allergen exposure, acute infections, medication changes, and patient adherence could all drive instability in phenotype.

The ADEPT (**A**irways **D**isease **E**ndotyping for **P**ersonalized **T**herapeutics) study profiled clinical characteristics and biomarkers in mild, moderate and severe asthma compared to healthy non-atopic controls, with a view to identifying clinical and biological phenotypes. The study design and population characteristics have been previously reported in detail [8].

Accordingly, an important objective of ADEPT was to follow mild, moderate and severe asthma subjects over 1 year to determine the stability of identified clinical and biological phenotypes, a period which would cover seasonal changes (allergen, climate, infection) and also allow spontaneous variability. Herein, we report the clinical and biomarker characteristics and how these varied over the 12-month course of the study.

## Methods

The study received institutional ethics approval at all sites. All subjects provided written informed consent to participate. The clinicaltrials.gov identifier is NCT01274507. A complete description of the study design, recruited population and disease characteristics has been previously reported in detail [8].

### Population

Approximately 150 asthma subjects (50 subjects in each of 3 asthma categories (mild, moderate, severe) were planned for inclusion in the study. The National Heart, Lung, and Blood Institute (NHLBI) expert panel report [9] was adapted for classification of severity based on lung function and controller medication levels. All subjects were non-smokers for ≥ 1 year at the initial screening visit and had ≤ 10 pack-year history of smoking.

Severity of asthma was defined at screening based on the level of background medications and lung function as previously reported [8]. Briefly, 52 mild (no asthma controller medications, forced expired volume in 1 s (FEV1) > 80 % predicted), 52 moderate (low-moderate dose inhaled corticosteroid (ICS), FEV1 between 60 to <80 % predicted), and 55 severe (high-dose ICS, FEV1 between 50 to <80 % predicted) asthma subjects were enrolled. The definition of low, medium and high ICS levels was based on the National Institutes of Health Clinical Practice Guidelines: Expert Panel Report 3: Guidelines for the Diagnosis and Management of Asthma., 2007 [9].

### Study design and visits

Detailed study design and methodologies have previously been reported in detail but a brief summary is provided below [8].

Asthma subjects underwent screening, then if enrolled attended the baseline visit. Further clinical assessment/biomarker visits occurred at 3, 6 and 12 months, with induced sputum sampling repeated at the 6 month visit. These 3, 6 and 12 month visits were intended to evaluate variability over time including the impact of seasons. During the study, investigators or treating physicians were permitted to make adjustments to asthma medications as medically needed.

### Clinical and biomarker assessments

Assessments measured longitudinally included pre-bronchodilator (pre-BD) and post-bronchodilator (post-BD) spirometric variables (FEV1, FVC, PEFR, and reversibility), the 7-item asthma control questionnaire ACQ7 [10], asthma quality of life questionnaire (AQLQ) [11], FENO, and induced sputum inflammatory cells. Detailed methods have been previously reported [8].

### Induced sputum

The method for induction and processing of sputum using the plug selection method has been described previously [12, 13]. Briefly, all study participants underwent induction for 21 min divided into three 7-min sessions of using an aerosol of hypertonic saline (in increasing concentrations of 3, 4, and 5 %). For those with FEV_1_ ≥ 50– < 60 % predicted, induction was performed with normal/isotonic saline (0.9 %). Subjects with post-BD FEV_1_ < 50 % predicted were not induced.

The plug selection method was used for this study with plugs treated with dithiothreitol. The detailed methodology has been previously reported [8].

### Statistical considerations

Significance of differences between groups was evaluated using General Linear Model analyses, using log-transformed data when necessary to satisfy assumption of normality of distributions (for FENO and sputum eosinophils, specifically). Correlations between variables were evaluated by calculation of the Pearson correlation coefficient (r).

Within-subject correlations over longitudinal visits for the clinical and clinical biomarker assessments were summarized via the intraclass correlation coefficient (ICC) (CRAN-R-project software (https://CRAN.R-project.org, version 3.2) package ‘ICC’ version 2.2.1). Confidence intervals (95 %) were estimated using the exact confidence limit equations appropriate for unbalanced data (THD option: [14]). The number of measurements per subject used in the ICC estimation is reported based on the equations from Lessells and Boag [15]. Box-and-whisker plot representations of distributions in figures show the median and interquartile range (box), minimum and maximum range (whiskers), mean (‘+’ symbol), and diamond symbols the values for each individual subject/sample.

## Results

The study enrolled 52 mild, 55 moderate, and 51 severe asthma subjects in North America and Europe from 2010–2013.

### Disposition of subjects in study

The retention of subjects was high in ADEPT as shown in Table 1. Seventeen of 158 asthma subjects withdrew prematurely (1 for a non-serious adverse event (AE); 5 withdrew consent, 2 for pregnancy, 4 for sponsor decisions, 2 were lost to follow-up and 3 withdrew for other reasons) as previously detailed [8].

**Table 1:** Retention of ADEPT asthma subjects in study

	Number of subjects				% of number of subjects at baseline			
	Baseline	Month 3	Month 6	Month 12	Baseline	Month 3	Month 6	Month 12
Mild	52	48	47	45	100 %	92 %	90 %	87 %
Moderate	55	50	50	49	100 %	91 %	91 %	89 %
Severe	51	48	47	47	100 %	94 %	92 %	92 %
Total	158	146	144	141	100 %	92 %	91 %	89 %

### Stability of background medications

Changes in background medications may reflect changes in asthma control but can also impact longitudinal stability, however few subjects had changes as detailed below.

#### Oral corticosteroids (OCS)

Two severe asthma subjects were on OCS during screening and at the baseline visit; one discontinued OCS at day 27 post-baseline whereas the second continued OCS throughout the study. Only a minority of subjects received OCS bursts during the study (4/51 mild (7.8 %), 3/55 moderate (5.5 %) and 8/51 severe (15.7 %) asthma subjects), which was greater for mild, but reduced for moderate and severe asthma subjects compared to the year prior to entry into the study (4 % of mild, 15 % of moderate, and 27 % of severe asthma).

#### Inhaled corticosteroids

Two mild asthma subjects were on ICS at baseline: 1 started ICS after screening while another reported taking ICS during screening. Three mild subjects started ICS post-baseline and 3 moderate subjects increased ICS dose post-baseline visit.

### Within-subject, longitudinal variation of asthma characteristics

Baseline characteristics have been previously reported [8], with the baseline values for the longitudinally assessed variables presented in Table 2.

**Table 2:** Asthma disease characteristics by cohort at baseline visit

	Asthma severity cohort			
Cohorts*	Mild	Moderate	Severe	P-value ***
N (total/sputum)	52/32	55/38	51/40	
Age (years)	33.7 (13.1)	45.0 (11.6)	46.2 (12.1)	<10^−6^
Pre-BD FEV1 (% predicted)	92.7 (14.3)	73.6 (10.4)	65.4 (12.7)	< 10^−6^
Pre-BD FEV1/FVC ratio	0.77 (0.08)	0.66 (0.09)	0.61 (0.09)	< 10^−6^
Post-BD FVC (% predicted)	105.0 (15.5)	96.4 (11.4)	94.0 (15.1)	0.0004
BDR (% change in FEV1)	8.5 (8.3)	15.2 (10.3)	18.3 (14.5)	0.0016
BDR (mL change in FEV1)	265.1 (231.7)	335.2 (234.3)	355.7 (270.6)	0.45
ACQ7	0.84 (0.69)	1.33 (0.71)	1.92 (1.01)	< 10^−6^
AQLQ	5.86 (0.93)	5.68 (1.11)	5.09 (1.28)	0.0016
FENO (ppb) **	32.9 (+64.2/−16.9)	29.1 (+61.0/−13.9)	28.8 (+64.7/−12.9)	0.59
Sputum eosinophils, % of WBC **	1.12 (+5.38/−0.93)	3.12 (+13.62/−2.54)	2.70 (+12.27/−2.21)	0.033
Sputum lymphocytes, % of WBC	1.29 (1.42)	0.98 (1.19)	0.94 (1.25)	0.48
Sputum macrophages, % of WBC	50.20 (31.17)	32.10 (21.45)	43.04 (26.46)	0.019
Sputum neutrophils, % of WBC	43.88 (30.90)	56.99 (26.13)	48.10 (25.52)	0.13

* Mean (standard deviation) reported by cohort, unless otherwise indicated

** Geometric mean (asymmetric standard deviation) reported by cohort

*** p-value (ANOVA F-test for differences across asthma severity cohorts (based on log-transformed data when geometric means reported)

**Fig. 1:**
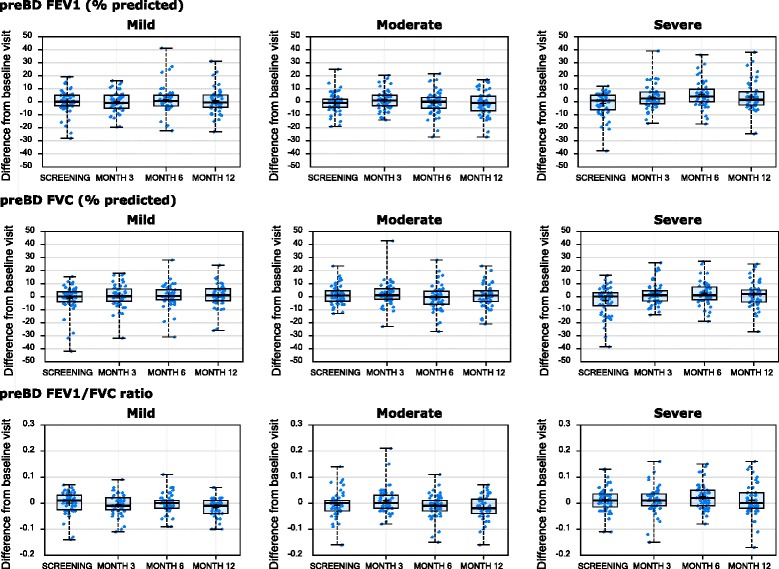
Longitudinal stability of lung function parameters. Changes from baseline visit (y-axis) for the indicated clinical variable (at top of row of plots) are shown by visit (x-axis), stratified by asthma severity cohort (indicated at top of plot)

**Fig. 2:**
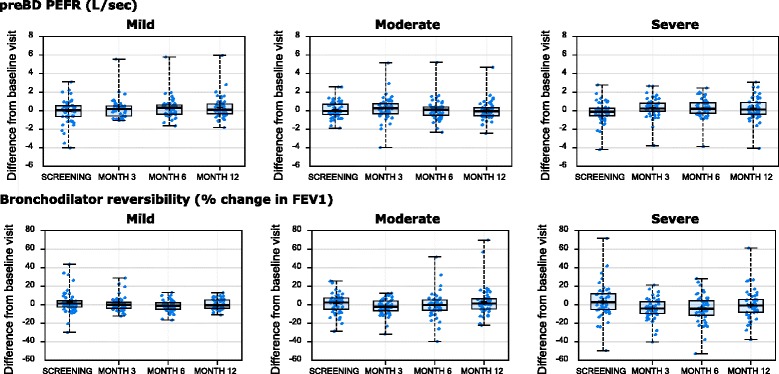
Longitudinal stability of lung function parameters. Changes from baseline visit (y-axis) for the indicated clinical variable (at top of row of plots) are shown by visit (x-axis), stratified by asthma severity cohort (indicated at top of plot)

**Fig. 3:**
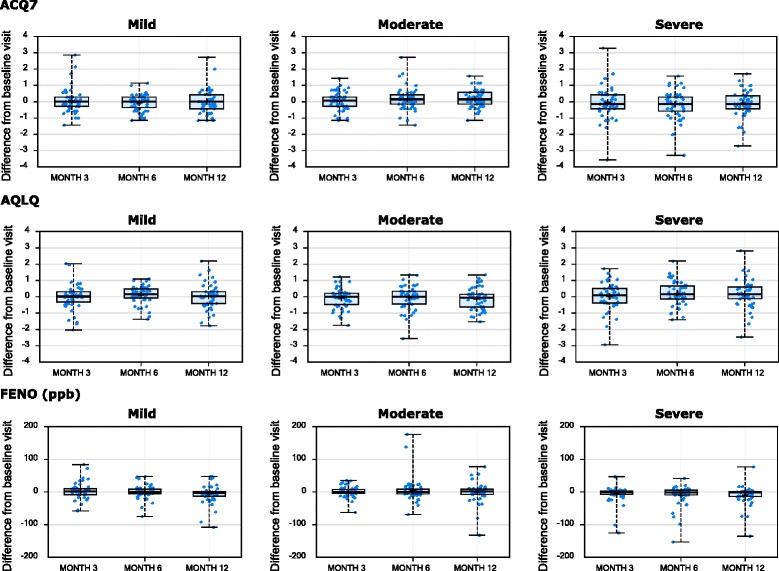
Longitudinal stability of patient reported outcomes and FENO. Changes from baseline visit (y-axis) for the indicated clinical variable (at top of row of plots) are shown by visit (x-axis), stratified by asthma severity cohort (indicated at top of plot)

For the longitudinally assessed clinical characteristics, mean values did not significantly vary over the course of the study (data not shown). However, there was obvious intra-individual variability across the visits, with the within-subject changes from the baseline visit for the longitudinally assessed characteristics presented in Figs. 1, 2 and 3. This intra-individual variability was statistically summarized by the ICC method (see Table 3). There was no consistent difference in variability by severity.

**Table 3:** Intraclass correlation coefficients for clinical characteristics over all study visits

ICC (95 % CI) [number of visits]	Mild (*n* = 52)	Moderate (*n* = 55)	Severe (*n* = 51)	All asthma (*n* = 158)
FEF_2575_, pre-BD	0.80 (0.73–0.87) [4.5]	0.76 (0.68–0.84) [4.6]	0.67 (0.56–0.77) [4.7]	0.82 (0.78–0.86) [4.6]
FEV1/FVC, pre-BD	0.86 (0.80–0.91) [4.5]	0.79 (0.71–0.86) [4.6]	0.84 (0.77–0.89) [4.7]	0.88 (0.85–0.90) [4.6]
FEV1 % predicted, pre-BD	0.72 (0.62–0.81) [4.5]	0.60 (0.48–0.71) [4.6]	0.64 (0.52–0.75) [4.7]	0.81 (0.77–0.85) [4.6]
FVC % predicted, pre-BD	0.79 (0.71–0.86) [4.5]	0.62 (0.51–0.73) [4.5]	0.75 (0.66–0.83) [4.7]	0.79 (0.74–0.83) [4.6]
PEFR, pre-BD	0.83 (0.76–0.89) [4.5]	0.81 (0.73–0.87) [4.6]	0.86 (0.80–0.91) [4.7]	0.86 (0.83–0.89) [4.6]
FEV1 change post-BD (% predicted)	0.45 (0.32–0.59) [4.5]	0.39 (0.26–0.53) [4.5]	0.39 (0.26–0.54) [4.6]	0.42 (0.35–0.50) [4.5]
FEV1 change post-BD (L)	0.45 (0.32–0.59) [4.5]	0.53 (0.41–0.66) [4.5]	0.47 (0.34–0.61) [4.6]	0.48 (0.41–0.56) [4.5]
FENO, ppb (log)	0.64 (0.51–0.75) [3.6]	0.78 (0.69–0.86) [3.6]	0.75 (0.65–0.84) [3.6]	0.73 (0.67–0.78) [3.6]
ACQ-7	0.61 (0.48–0.73) [3.6]	0.57 (0.43–0.69) [3.6]	0.55 (0.41–0.69) [3.7]	0.65 (0.58–0.71) [3.6]
AQLQ	0.74 (0.63–0.82) [3.6]	0.82 (0.74–0.88) [3.6]	0.78 (0.68–0.85) [3.7]	0.79 (0.74–0.83) [3.6]

### Longitudinal stability of lung function

Pre-BD FEV1/FVC ratio and PEFR showed the highest ICC values (>0.79) across all severities reflecting low variability. Percent predicted Pre-BD FEV1 and FVC had lower ICC values (0.60–0.75) for moderate and severe asthma cohorts.

### Longitudinal stability of bronchodilator response

Bronchodilator reversibility (BDR) was assessed at screening and at every study visit. BDR expressed as % change or absolute change had low ICC values for cross-visit correlations ranging between 0.39–0.45 across severities, reflecting low stability of this measure (see Table 3).

**Fig. 4:**
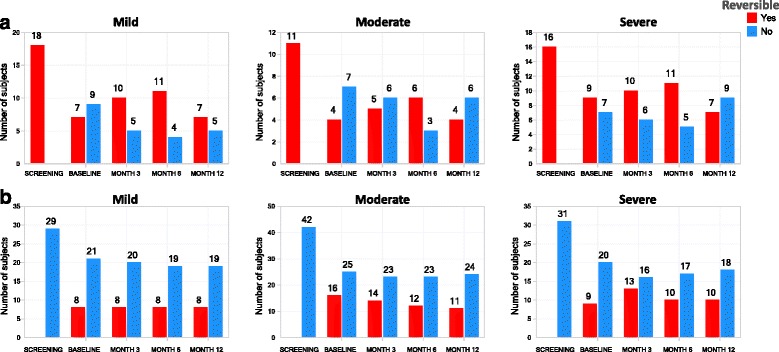
Longitudinal stability of the bronchodilator reversible phenotype. The number of subjects (y-axis, number label) with reversible (*red bars*) and non-reversible (*blue bars*) phenotypes for response to bronchodilator, based on cut-off of change in FEV1 of 12 % and 200 ml, at the indicated visit (x-axis) are shown for subjects that were reversible (**a**) or non-reversible (**b**) at the screening visit, stratified by asthma severity cohort

Defining a reversible phenotype as a change from baseline of ≥12 % and ≥200mls, the reversible phenotype was highly variable across cohorts, with screening vs. baseline concordances of 44, 36, and 56 % for mild, moderate, and severe cohorts, respectively and variable concordance rates at the 3, 6, and 12 month visits (Fig. 4a). In contrast, the non-reversible phenotype was more stable than the reversible phenotype, with screening vs. baseline concordances of 72, 61, and 69 % for mild, moderate, severe cohorts, respectively and similar concordance rates seen at the 3, 6, and 12 month visits (Fig. 4b).

### Longitudinal stability of ACQ7

**Fig. 5:**
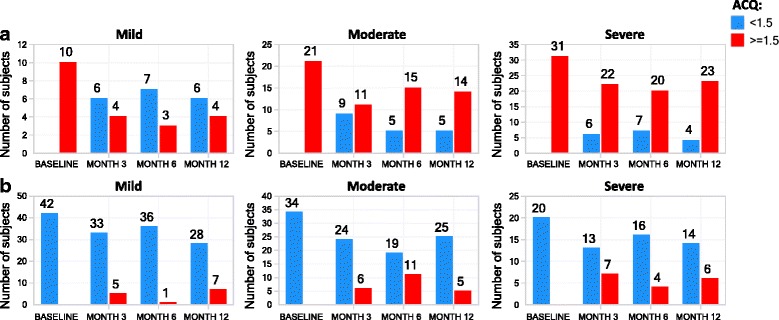
Longitudinal stability of asthma control phenotype. The number of subjects (y-axis, number label) with poorly controlled (ACQ ≥1.5, *red*) and controlled (ACQ < 1.5, *blue*) asthma phenotypes, at the indicated visit (x-axis) are shown for subjects with ACQ ≥ 1.5 (**a**) or ACQ < 1.5 (**b**) at the baseline visit, stratified by asthma severity cohort

ACQ was less stable (ICC 0.55–0.61) than AQLQ, spirometric measures, or FENO across asthma severities (Table 3). In mild asthma (non-ICS-treated), for those with an ACQ > 1.5 at baseline (uncontrolled asthma), there was considerable variability with only 40 % concordance at month 3 (Fig. 5a), in contrast to those with an ACQ < 1.5 at baseline (controlled asthma), with 87 % concordance at month 3, (Fig. 5b). For moderate asthma, there was considerable variability for the ACQ ≥ 1.5 phenotype at baseline (Fig. 5a), with only 55 % concordance at month 3, but 80 % concordance for those with a baseline ACQ < 1.5 at month 3 (Fig. 5b). In severe asthma, there was 79 % concordance at month 3 for ACQ ≥ 1.5 at baseline (Fig. 5a) but only 65 % concordance at month 3 with the baseline ACQ < 1.5 (Fig. 5b). Thus moderate asthmatic subjects showed a greater tendency to become controlled over time (ACQ < 1.5) compared to mild asthma while most severe asthmatics who were uncontrolled at baseline, remained uncontrolled later on. Similar observations pertain to months 6 and 12.

AQLQ showed greater stability over time compared to ACQ with ICC values in the 0.74 to 0.82 range, with mild asthma (who were non-ICS-treated) showing greater variability than moderate and severe asthma (Table 3).

### Longitudinal stability of FENO

FENO was measured at screening, and at all study visits. Longitudinal stability was moderate in mild asthma with mean (95 % CL) ICC values 0.64 (0.51–0.75), but higher in moderate 0.78 (0.69–0.86), and severe asthma 0.75 (0.65–0.84) (Table 3).

**Fig. 6:**
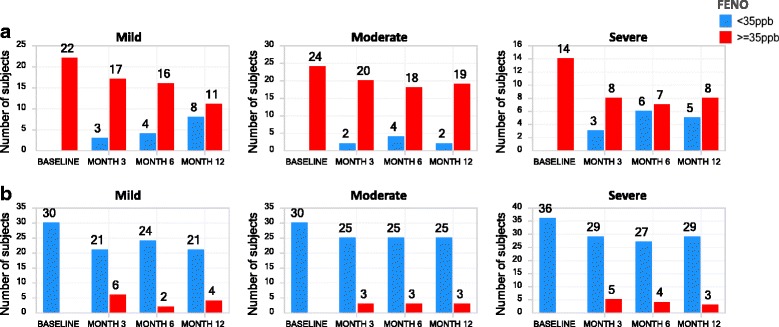
Longitudinal stability of FENO phenotypes. The number of asthma subjects (y-axis, number label) with FENO-high (≥35 ppb, red bars) and FENO-low (<35 ppb, blue bars) phenotypes, at the indicated visit (x-axis) are shown for subjects with FENO ≥ 35pbb (**a**) or FENO < 35 ppb (**b**) at the baseline visit, stratified by asthma severity cohort

Using 35 ppb as a cutoff to define high- and low-FENO [16] at baseline, the high-FENO phenotype showed reduced concordances for the severe asthma cohort at the month 3, 6 and 12 visits compared to the moderate cohort. Of note, there were 58 and 54 % concordances for mild asthma at month 12 and severe asthma at month 6, respectively. The moderate cohort maintained high concordances for the FENO-high phenotype, with the lowest concordance at month 6 (82 %). Overall, there were 85, 75, and 72 % concordances at months 3, 6, and 12, respectively, across the 3 severity cohorts combined for the FENO-high defined at baseline (Fig. 6a).

In contrast, the FENO-low phenotype at baseline was generally stable across severity cohorts at subsequent study visits. Mild asthma had the lowest concordance (77 %) at Month 3 for the FENO-low phenotype defined at baseline. In contrast, there were 84, 89, and 88 % concordances for the FENO-low phenotype at months 3, 6, and 12, respectively, across the 3 severity cohorts (Fig. 6b).

### Longitudinal stability of induced sputum inflammatory cells

Sputum was induced at screening (required for eligibility), baseline, and at the 6 month visit. There were 92, 73, and 55 asthma subjects with acceptable differential counts available at the screening, baseline, and Month 6 visits, respectively, with 117 subjects having acceptable counts for at least one visit. Of the 92 asthma subjects with acceptable screening visit counts, only 52 and 41 had acceptable counts at the baseline and month 6 visits, respectively.

**Fig. 7:**
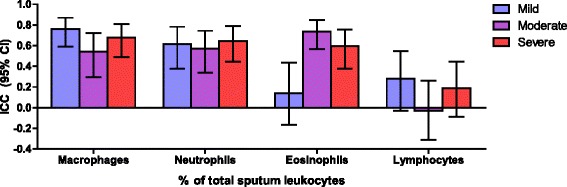
Longitudinal stability of sputum leukocyte subsets. Intraclass correlation coefficients (ICC) and 95 % confidence intervals are shown for the indicated sputum leukocyte subset (% of total sputum leukocytes; x-axis), stratified by asthma severity cohort

Seventy two asthma subjects had acceptable counts for at least 2 visits and 42 subjects for all 3 visits. For all asthma severities combined, the proportion of spMAC demonstrated the highest ICC across the screening, baseline, and month 6 measurements (0.71) followed by spNEU (0.63), and spEOS (0.58) (Fig. 7). The very low stability of spLYM was perhaps related to the very low proportions of this cell. When taking asthma severity into account, there was low stability of eosinophil proportions in mild asthma probably related to the low proportion of mild subjects with sputum eosinophilia.

Considering commonly-used definitions for 4 sputum phenotypes, paucigranulocytic (spNEU < 60 % and spEOS < 3 %), neutrophilic (spNEU ≥ 60 %), eosinophilic (spEOS ≥ 3 %) [17] and mixed inflammatory phenotypes (spNEU ≥ 60 % and spEOS ≥ 3 %), differing stability patterns were observed (Table 4). Comparing the baseline visit to screening (*n* = 52), concordance was highest for the neutrophilic phenotype (62 %), followed by the mixed (57 %), eosinophilic and paucigranulocytic phenotypes (both 50 %) (Table 4). Comparing the 6-month visit to screening (*n* = 41), concordance was highest for the paucigranulocytic (77 %) and eosinophilic (71 %) phenotypes, followed by the neutrophilic (43 %) and mixed phenotypes (29 %) (Table 5).

**Table 4:** Concordance for 4 sputum inflammatory cell phenotypes at baseline compared to screening

% of Screening	Screening: concordance for 4 inflammatory cell phenotypes			
Baseline	Eos < 3 %; PMN < 60 %	Eos < 3 %; PMN ≥ 60 %	Eos > =3 %; PMN ≥ 60 %	Eos ≥ 3 %; PMN < 60 %
Eos < 3 %; PMN < 60 %	50 %	24 %	0 %	19 %
Eos < 3 %; PMN ≥ 60 %	33 %	62 %	29 %	25 %
Eos ≥ 3 %; PMN ≥ 60 %	0 %	10 %	57 %	6 %
Eos ≥ 3 %; PMN < 60 %	17 %	5 %	14 %	50 %
% of Screening	Screening: concordance for 2 inflammatory phenotypes			
Baseline	Eos < 3 %	Eos ≥ 3 %	PMN < 60 %	PMN ≥ 60 %
Eos < 3 %	84 %	39 %		
Eos ≥ 3 %	16 %	61 %		
PMN < 60 %			68 %	25 %
PMN ≥ 60 %			33 %	75 %

**Table 5:** Concordance for the 4 and 2 sputum inflammatory cell phenotypes at 6 months compared to screening

% of Month 6	Month 6 concordance for 4 sputum inflammatory cell phenotypes			
Screening	Eos < 3 %; PMN < 60 %	Eos < 3 %; PMN ≥ 60 %	Eos ≥ 3 %; PMN > =60 %	Eos ≥ 3 %; PMN < 60 %
Eos < 3 %.; PMN < 60 %	77 %	8 %	15 %	0 %
Eos < 3 %; PMN ≥ 60 %	43 %	43 %	14 %	0 %
Eos ≥ 3 %; PMN ≥ 60 %	0 %	57 %	29 %	14 %
Eos ≥ 3 %; PMN < 60 %	21 %	0 %	7 %	71 %
% of Month 6	Month 6 concordance for 2 sputum inflammatory cell phenotypes			
Screening	Eos < 3 %	Eos ≥ 3 %	PMN < 60 %	PMN ≥ 60 %
Eos < 3 %	85 %	15 %		
Eos ≥ 3 %	33 %	67 %		
PMN < 60 %			85 %	15 %
PMN ≥ 60 %			29 %	71 %

Longitudinal stability was also assessed sub-setting on only spNEU (NEU-high = spNEU ≥ 60 % vs. NEU-low = spNEU <60 %) or only spEOS (EOS-high = spEOS ≥ 3 % vs. EOS-low = spEOS < 3 %) phenotypes. Comparing baseline to screening, concordance was highest for the EOS-low (84 %) and NEU-high (75 %) phenotypes, and moderate for the NEU-low (68 %) and EOS-high (61 %) phenotypes (Table 4). Comparing 6-month to screening measurements (*n* = 41), concordance was highest for the EOS-high (85 %) and NEU-low (85 %) phenotypes, and moderate for the NEU-high (71 %) and EOS-high (67 %) phenotypes (Table 5).

Appreciating the uncertainty in the appropriate cut-off value for sputum neutrophils relating to pathology in asthma, additional cut-off values were tested. Screening-to-baseline visit concordance for neutrophil high/low phenotypes using 40, 60, and 80 % cut-offs were 75, 71, and 80 %, respectively. The concordances for screening-to-month 6 visit were 87, 84, and 81 %, respectively. Also, the ICC for proportion of neutrophils over the 3 visits, which does not require a cut-off to be specified, demonstrated moderate stability (ICC = 0.63, 95 % CI: 0.52–0.72).

## Discussion

The ADEPT study profiled mild, moderate, and severe asthma subjects and accrued biomarker data across multiple matrices in the majority of subjects and evaluated several of these matrices repeatedly over 12 months. Of note, while most clinical and biomarker characteristics were stable in the severity cohorts as a whole, there was marked individual variability in most parameters.

For lung function, pre-BD FEV1/FVC ratio and PEFR showed the best reproducibility, while pre-BD FEV1 and FVC % predicted were more variable possibly reflecting fluctuations in asthma control, in keeping with the inherent variability of airway smooth muscle contraction in asthma.

One of the most interesting findings of the present study is the poor reproducibility of the BDR over a 12-months period. The factors underlying this variability likely include spontaneous change in asthma control, and patient adherence as well as technical factors including the withholding of bronchodilators before testing as dictated by the protocol, the time of the BDR (due to circadian variability) and inherent variability in the spirometry test itself.

Moreover, there was low concordance from visit to visit in terms of defining a reversible phenotype. The vast majority of clinical trials in asthma require demonstration of BDR for eligibility predominantly due to regulatory requirements to objectively “confirm” the diagnosis of asthma, although COPD can demonstrate significant reversibility as well [18]. This requirement undoubtedly excludes a substantial number of subjects with asthma from studies. Moreover, it appears that reversibility is not a stable phenotype and perhaps eligibility criteria for studies could be relaxed in this regard, if other features of asthma were present, historical reversibility documented, or AHR demonstrated. Similarly, the spontaneous variation in both FEV1 and BDR even in severe asthma suggests that low FEV1 should not necessarily be a prerequisite for inclusion of patients into severe asthma studies.

In general, the ACQ only demonstrated moderate stability across severities which may be related to the inherent variability in asthma driven by seasonal exposures, climatic factors, and infections. The controlled phenotype (ACQ < 1.5) was more stable in mild and moderate asthma than in severe asthma while these 2 cohorts showed less stability for the uncontrolled phenotype (ACQ ≥ 1.5). This suggests that lack of control in mild and moderate asthma was not always a persistent state. In severe asthma, those who were uncontrolled in general remained so, perhaps reflecting the persistent severity of the disease despite high-dose inhaled corticosteroid therapy, and in many cases a lack of appropriate targeted therapy for this challenging group of patients.

FENO and the FENO-high categorization both showed reasonable stability in moderate and severe asthma suggesting that the FENO-high phenotype is a useful phenotype and could be used reliably to select patients for therapeutics as has been reported with ICS therapy [19] and other type-2 therapies [1]. In the mild asthma cohort, whose subjects were not treated with ICS during the study, FENO-high concordance was slightly lower perhaps due to the lack of anti-inflammatory therapy. Others have also found FENO to be reasonably stable over time [20–22]. Of note, a high proportion of patients had consistently low FENO, perhaps indicating that non-eosinophilic asthma was common.

When evaluating four sputum inflammatory phenotypes, the picture that emerges is one of inherent variability in agreement with the findings of Al-Samri et al [23], thus casting doubt as to whether these phenotypes will prove to be useful long-term patient-selection strategies. Thus comparing baseline to screening, the neutrophilic phenotype was the most stable however when comparing 6-month to screening-BL, the eosinophilic phenotype was the most stable. Reducing the number of sputum phenotypes to two, namely EOS with or without NEU, or NEU with or without EOS, the ranking of the concordances showed no consistent pattern. Reasons for this could include spontaneous variability, seasonal change, climate change, patient adherence, intercurrent infections, as well as technical issues affecting sample quality.

Others have reported on the stability or lack thereof of clinical and biomarker characteristics in asthma. Blood eosinophils were only measured during screening in ADEPT but published data suggest substantial variability even during a 24 h period [24] again raising the question as to whether eosinophilic asthma, defined by bEOS, is a stable phenotype. Fleming et al reported low reproducibility for sputum eosinophils in children [25] as did Rossall et al in adults with moderate to severe asthma [26]. In contrast Simpson et al found these measures to be stable in adults with stable asthma [27].

The variability seen in ADEPT and in other studies for the profile of inflammatory cells in sputum probably reflects similar factors that contribute to variability in asthma e.g. allergen exposure, viral infections and technical issues with sputum induction. Thus, seasonal allergen exposure could enhance Type 2 inflammation resulting in greater eosinophilic inflammation and elevations in FENO as previously described. Viral infections, which also have a seasonal pattern, could drive neutrophilic inflammation that could persist after clinical resolution.

Due to protocol restrictions for safety reasons in those undergoing bronchoscopy, the ADEPT asthma cohort did not include subjects with a BMI > 32 kg/m^2^, those >65 years of age, current smokers and those on oral corticosteroids. The findings may therefore not be applicable in their entirety to severe refractory asthma.

## Conclusions

In summary, the ADEPT asthma cohort presented a unique opportunity to follow clinical status and biomarkers in a well-characterized cohort over 12 months. ADEPT confirms substantial variability in clinical characteristics but importantly in clinical biomarkers as well suggesting, that biological phenotypes are highly variable in some patients, perhaps related to the same seasonal changes that drive clinical variability. The implications for using biomarkers assessed at a single time point may be profound.

## Abbreviations

ACQ7: asthma control questionnaire 7 (includes FEV1)
ADEPT: Airways Disease Endotyping for Personalized Therapeutics study
AHR: airways hyperresponsiveness
AQLQ: asthma quality of life questionnaire
BDR: bronchodilator reversibility
bEOS: blood eosinophils
COPD: chronic obstructive pulmonary disease
CRF: case report form
EOS: eosinophils
EPBR: epithelial brushing
ERS: European Respiratory Society
FEF25-75: forced expired flow averaged between 25–75 % of the vital capacity
FENO: fractional concentration of exhaled nitric oxide
FEV1: forced expired volume in 1 second
FEV1: forced expired volume in 1 second
FVC: forced vital capacity
ICC: intraclass correlation coefficient
ICS: inhaled corticosteroid
IgE: immunoglobulin E
IL: interleukin
IS: induced sputum
LABA: long-acting β-2 agonist
mAb: monoclonal antibody
ml: milliliters
NA: not applicable
NEU: neutrophilic
OCS: oral steroids
PC20: the provocative concentration of methacholine resulting in a 20 % or greater fall in the forced expired volume in 1 second
PEFR: peak expiratory flow rate
Post-BD: post-bronchodilator
ppb: parts per billion
Pre-BD: pre-bronchodilator
SABA: short-acting β-2 agonist
sIgE: serum immunoglobulin E
spEOS: sputum eosinophils
spLYM: sputum lymphocytes
spMAC: sputum macrophages
spNEU: sputum neutrophils
Th2: T helper cell type 2
WBC: white blood cells

## References

[1] Corren J, Lemanske RF, Hanania NA, Korenblat PE, Parsey MV, Arron JR, Harris JM, Scheerens H, Wu LC, Su Z (2011). Lebrikizumab treatment in adults with asthma. N Engl J Med.

[2] Brightling CE, Chanez P, Leigh R, O'Byrne PM, Korn S, She D, May RD, Streicher K, Ranade K, Piper E (2015). Efficacy and safety of tralokinumab in patients with severe uncontrolled asthma: a randomised, double-blind, placebo-controlled, phase 2b trial. Lancet Respir Med.

[3] Bel EH, Wenzel SE, Thompson PJ, Prazma CM, Keene ON, Yancey SW, Ortega HG, Pavord ID, Investigators S (2014). Oral glucocorticoid-sparing effect of mepolizumab in eosinophilic asthma. N Engl J Med.

[4] Haldar P, Brightling CE, Hargadon B, Gupta S, Monteiro W, Sousa A, Marshall RP, Bradding P, Green RH, Wardlaw AJ, Pavord ID (2009). Mepolizumab and exacerbations of refractory eosinophilic asthma. N Engl J Med.

[5] Pavord ID, Korn S, Howarth P, Bleecker ER, Buhl R, Keene ON, Ortega H, Chanez P (2012). Mepolizumab for severe eosinophilic asthma (DREAM): a multicentre, double-blind, placebo-controlled trial. Lancet.

[6] Castro M, Wenzel SE, Bleecker ER, Pizzichini E, Kuna P, Busse WW, Gossage DL, Ward CK, Wu Y, Wang B (2014). Benralizumab, an anti-interleukin 5 receptor alpha monoclonal antibody, versus placebo for uncontrolled eosinophilic asthma: a phase 2b randomised dose-ranging study. Lancet Respir Med.

[7] Hanania NA, Wenzel S, Rosen K, Hsieh HJ, Mosesova S, Choy DF, Lal P, Arron JR, Harris JM, Busse W (2013). Exploring the effects of omalizumab in allergic asthma: an analysis of biomarkers in the EXTRA study. Am J Respir Crit Care Med.

[8] Silkoff PE, Strambu I, Laviolette M, Singh D, FitzGerald JM, Lam S, Kelsen S, Eich A, Ludwig-Sengpiel A, Hupp GC (2015). Asthma characteristics and biomarkers from the Airways Disease Endotyping for Personalized Therapeutics (ADEPT) longitudinal profiling study. Respir Res.

[9] National Asthma E, Prevention P (2007). Expert Panel Report 3 (EPR-3): Guidelines for the Diagnosis and Management of Asthma-Summary Report 2007. J Allergy Clin Immunol.

[10] Juniper EF, O'Byrne PM, Guyatt GH, Ferrie PJ, King DR (1999). Development and validation of a questionnaire to measure asthma control. Eur Respir J.

[11] Juniper EF, Buist AS, Cox FM, Ferrie PJ, King DR (1999). Validation of a standardized version of the Asthma Quality of Life Questionnaire. Chest.

[12] Pin I, Gibson PG, Kolendowicz R, Girgis-Gabardo A, Denburg JA, Hargreave FE, Dolovich J (1992). Use of induced sputum cell counts to investigate airway inflammation in asthma. Thorax.

[13] Kelly MM, Efthimiadis A, Hargreave FE (2001). Induced sputum : selection method. Methods Mol Med.

[14] Thomas JD, Hultquist RA (1978). Interval Estimation for the Unbalanced Case of the One-Way Random Effects Model.

[15] Lessells CM, Boag PT (1987). Unrepeatable repeatabilities: a common mistake. Auk.

[16] Dweik RA, Sorkness RL, Wenzel S, Hammel J, Curran-Everett D, Comhair SA, Bleecker E, Busse W, Calhoun WJ, Castro M (2010). Use of exhaled nitric oxide measurement to identify a reactive, at-risk phenotype among patients with asthma. Am J Respir Crit Care Med.

[17] Korevaar DA, Westerhof GA, Wang J, Cohen JF, Spijker R, Sterk PJ, Bel EH, Bossuyt PM (2015). Diagnostic accuracy of minimally invasive markers for detection of airway eosinophilia in asthma: a systematic review and meta-analysis. Lancet Respir Med.

[18] Celli BR, Tashkin DP, Rennard SI, McElhattan J, Martin UJ (2011). Bronchodilator responsiveness and onset of effect with budesonide/formoterol pMDI in COPD. Respir Med.

[19] Little SA, Chalmers GW, MacLeod KJ, McSharry C, Thomson NC (2000). Non-invasive markers of airway inflammation as predictors of oral steroid responsiveness in asthma. Thorax.

[20] Kharitonov SA, Gonio F, Kelly C, Meah S, Barnes PJ (2003). Reproducibility of exhaled nitric oxide measurements in healthy and asthmatic adults and children. Eur Respir J.

[21] Ekroos H, Karjalainen J, Sarna S, Laitinen LA, Sovijarvi AR (2002). Short-term variability of exhaled nitric oxide in young male patients with mild asthma and in healthy subjects. Respir Med.

[22] Thijs W, de Mutsert R, le Cessie S, Hiemstra PS, Rosendaal FR, Middeldorp S, Rabe KF (2014). Reproducibility of exhaled nitric oxide measurements in overweight and obese adults. BMC Res Notes.

[23] Al-Samri MT, Benedetti A, Prefontaine D, Olivenstein R, Lemiere C, Nair P, Martin JG, Hamid Q (2010). Variability of sputum inflammatory cells in asthmatic patients receiving corticosteroid therapy: A prospective study using multiple samples. J Allergy Clin Immunol.

[24] Spector SL, Tan RA (2012). Is a single blood eosinophil count a reliable marker for "eosinophilic asthma?". J Asthma.

[25] Fleming L, Tsartsali L, Wilson N, Regamey N, Bush A (2012). Sputum inflammatory phenotypes are not stable in children with asthma. Thorax.

[26] Rossall MR, Cadden PA, Molphy SD, Plumb J, Singh D (2014). Repeatability of induced sputum measurements in moderate to severe asthma. Respir Med.

[27] Simpson JL, McElduff P, Gibson PG (2010). Assessment and reproducibility of non-eosinophilic asthma using induced sputum. Respiration.

